# Intratumor Heterogeneity as a Prognostic Factor in Solid Tumors: A Systematic Review and Meta-Analysis

**DOI:** 10.3389/fonc.2021.744064

**Published:** 2021-10-15

**Authors:** Tao Yu, Xin Gao, Zicheng Zheng, Xinyu Zhao, Shiyao Zhang, Chunqiang Li, Gang Liu

**Affiliations:** ^1^ Department of Oncology, Tianjin Medical University General Hospital, Tianjin, China; ^2^ Department of General Surgery, Tianjin Medical University General Hospital, Tianjin, China; ^3^ Institute of General Surgery, Tianjin Medical University General Hospital, Tianjin, China

**Keywords:** intratumor heterogeneity, solid tumors, prognosis, clinical prognosis index, meta-analysis

## Abstract

**Background:**

The landscape of intratumor heterogeneity (ITH) is present from the tumor evolution. ITH is a promising clinical indicator, but the association between ITH and prognosis remains controversial. Therefore, a meta-analysis was performed to explore whether ITH can serve as a valuable prognostic indicator in solid tumors.

**Methods:**

All included studies were from PubMed, Embase, Cochrane, and Web of Science databases up to October 10, 2020. Studies based on ITH with available prognostic information were included. Three researchers independently completed study selection and data extraction following PRISMA guidelines. The random-effect model was used for synthesis. Hazard ratio (HR) and 95% confidence intervals (CI) were used with the endpoint defined by overall survival (OS), disease-specific survival (DFS), and progression-free survival (PFS).

**Results:**

A total of 9,804 solid tumor patients from 21 studies were included. Analysis of specific cancers in the TCGA database showed similar results based on different ITH assessment methods, which provided the logical support for data consolidation. Available evidence revealed a negative relationship between ITH and prognosis for a specific cancer (such as lung cancer). However, the OS results from 14 tumor types showed that high ITH associated with shorter survival time [HR 1.65 (95% CI, 1.42–1.91)]. PFS and DFS analyses showed similar results [HR 1.89 (95% CI, 1.41–2.54) and HR 1.87 (95% CI, 1.15–3.04)] in general. The status of tumor metastasis and sampling models were not the confounding factors.

**Conclusions:**

High ITH is associated with worse prognosis in many solid tumors in general although this association was absent for some cancers. ITH is expected to be a promising clinical prognostic factor for the improvement of assessment, treatment, and surveillance strategy.

## Introduction

In 1976, Peter C. Nowell (1) laid the foundation for the clonal evolution model of cancer. He hypothesized that tumors arise from a founder cell and that genetic instability gives the tumor the potential to produce multiple “sublines.” In 1977, Fidler et al. (2) inoculated different parts of tumor cells of melanoma cells into mice and produced a variety of lung metastases numbers and proposed that there are distinct “clones” in tumors. In recent years, with the rapid development of next-generation sequencing technology, intratumor heterogeneity (ITH) reflects the subclone information of solid tumors and is used to study tumor metastatic patterns (3–9). In addition, ITH has the potential to be a new clinical prognostic indicator (10, 11). However, the results of previous studies did not provide consistent conclusion regarding the association between ITH and prognosis.

ITH is the genetic diversity reserve of tumors in the face of natural selection, which is largely responsible for the failure of targeted therapies (12). However, a key question is whether this diversity has important impact on a tumor’s natural history or a patient’s outcome (especially those who do not receive targeted therapy). To clarify these issues, we conducted a meta-analysis to explore the relationship between ITH and the prognosis of patients with solid tumors and evaluate the ability of ITH in predicting outcome by stratified confounding factors.

## Methods

We performed a systematic review and meta-analysis following PRISMA guidelines (registration numbers CRD42020193878 and INPLASY202060107).

We retrieved studies from PubMed, Embase, Cochrane, and Web of Science databases up to October 10, 2020, using ITH and prognosis as the main keywords. We also screened EMSO, ASCO, and WCLC of recent years to avoid missing updated data. The type of study or language of the study was not limited in the study collection.

### Inclusion Criteria

The inclusion criteria were as follows:

1. The study focused on solid tumors and examined ITH.
2. The study adopted a complete system for the assessment of ITH with the following steps: sample acquisition, extraction of genetic information, and evaluation of ITH by a scientific algorithm.
3. The study provided clear prognostic information and retrievable results.

### Outcome

All studies included prognostic endpoints including overall survival (OS), disease-specific survival (DFS), and progression-free survival (PFS).

### Exclusion Criteria

The exclusion criteria were as follows:

1. The source of the sample was not taken from the tumor region.
2. RNA-ITH was excluded because most data were based on DNA sequencing.
3. Prognostic information was not associated with ITH or could not be extracted.

### Screening and Extraction

All studies were reviewed separately by three investigators (ZZ, TY, and XG) according to inclusion and exclusion criteria. In cases in which the results were different, the case was discussed to obtain consensus. The information from the included studies was extracted in accordance with unified standards. When the included cohort was at risk of repeat statistics for the same patients, after considering the number of participants studied, the standardization of genetic information assessment, and the evaluation instruments of ITH, only the most relevant cohort was included in each forest map. Original data were extracted when a clear hazard ratio (HR) and 95% confidence interval (CI) was provided in included studies. If studies just provided the survival curve and the number of patients, the Engauge Tool and HR Calculations Spreadsheet were used to extract the HR and 95% CI (13). We standardized descriptions of the tumor types involved in the study according to the classification of tumor types in the TCGA database. Kaplan–Meier curves or univariate Cox regression data were selected whenever possible.

### Study Quality Assessment

We used the Guidelines for Assessing Quality in Prognostic Studies on the Basis of the Framework of Potential Biases in the study of deviation analysis (14). The procedure was evaluated independently by two researchers (ZZ and TY), and disputes were resolved by a third researcher.

### Statistical Methods

The characteristics of all included participants were collected. All extracted HR and 95% CI were combined by the R version 4.0.2 with Meta package version 4.13-0. The Random Effects Model was used for all forest maps. Results were obtained through the combined HR and 95% CI. Data heterogeneity is represented by *I^2^*, with *I^2^* ≥ 50 considered as high heterogeneity. Sensitivity analysis was performed to explore the effects of high heterogeneity. Subgroup analysis was used to reduce heterogeneity and analyze confounding factors. It was analyzed from the perspectives of OS, PFS, DFS, ITH assessment method, distant metastasis or not, sampling model, and so on. Funnel plots were generated for each forest map to assess publication offset.

## Results

**Figure 1:**
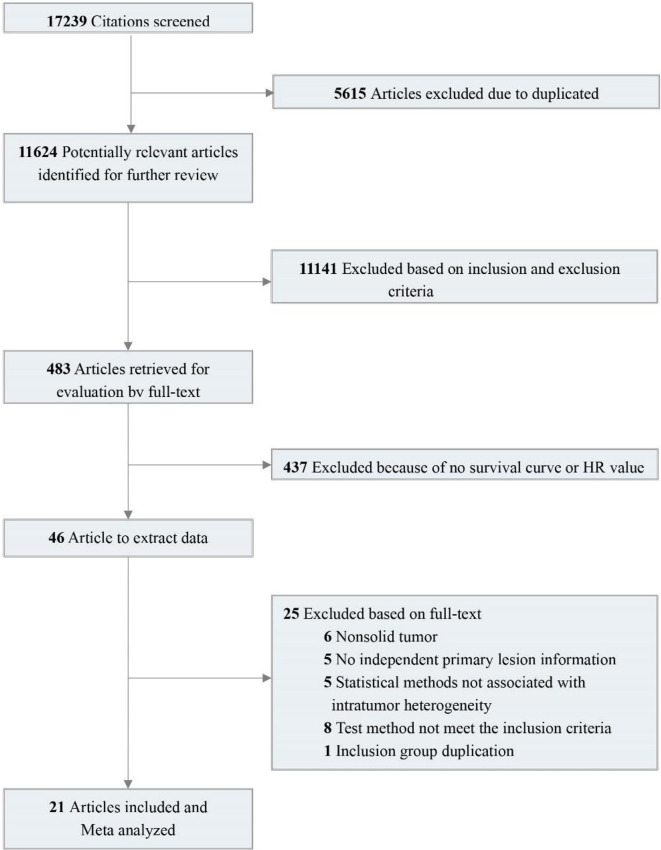
A flowchart of paper selection in our study.

A total of 17,239 relevant studies were retrieved. After removing duplicates, we screened 483 potentially relevant articles by scanning the titles and abstracts. We reviewed the full text and screened the candidate studies according to the inclusion criteria, and 437 studies with no prognostic information were excluded. Of the remaining 46 studies, 25 were excluded using the exclusion criteria. In the end, 21 studies were included (15–33). These studies contain 38 pieces of comparison information that were extracted. Details of the flow chart for study identification are shown in **Figure 1**.

Quality evaluation: The assessment includes study participation, study attrition, prognostic factor measurement, outcome measurement, confounding measurement and account, and analysis. Sixteen studies were not at risk in the quality assessment. Two studies were considered as high risk in the study participation, and four studies did not describe confounding measurement and account. No study had more than two risks (**Supplementary Table S1**).

Characteristics of all cohorts: The final study population included 9,804 participants. All studies are retrospective studies published between 2013 and 2020 and involved various tumor types (**Supplementary Table S2**).

### Different ITH Assessment Methods Had Limited Influence on This Study

Researchers have used a variety of methods for ITH evaluation and gradually formed some relatively stable platforms. Single-nucleotide variants (SNVs) and small indels are universally investigated DNA variations. The main ITH evaluation method is to calculate variant allele fraction (VAF) based on SNVs and small indel information. Some researchers (18, 19, 23, 30, 34) use VAF to calculate the number of clones by clone detection methods such as PyClone and EXPANDS. Other researchers (20, 22, 24, 25, 28, 31, 33, 35) directly use VAF to directly reflect the information of ITH through methods such as mutant-allele tumor heterogeneity (MATH). Copy number variations (CNVs), as genetic information with high mutation frequency, have also been recently used by a few researchers in the evaluation of ITH (26, 29). **Table 1** summarizes the details.

**Table 1:** Characteristics of ITH Assessment Methods.

Study (First author, year)	Cohort name	Detection	ITH assessment classification	ITH assessment details (cutoff)	Stage	Sampling	Participants(Low ITH)	Participants(High ITH)	HR (95% CI)
31	Chao-2020(STES)	SNV Array Panel	Based on clone numbers	Number of clones (Cutoff = 2)	I–III	Multi-region	22	19	3.92 (1.27,12.08)
32	Hou-2020(UCEC)	WES	Based on VAF directly	MATH (Cutoff: median)	I–IV	NA	121	121	2.34 (1.11,4.94)
15	Liu-2017(BLCA)-1	WES	Based on clone numbers	Number of clones (Cutoff = 6)	I–III	Single-region	15	15	1.64 (1.08,2.49)
16	Losic-2020(LIHC)	WES	Based on clone numbers	Number of clones (Cutoff = 4)	NA	NA	85	102	1.71 (1.19,2.44)
17	Mao-2019(LUAD)	WES	Based on VAF directly	MATH (Cutoff: median)	NA	NA	115	115	1.31 (0.86,2.00)
19	McDonald-2019(BRAC)	WES	Based on VAF directly	MATH (Cutoff: median)	I–IV	NA	411	548	1.36 (1.11,1.67)
20	Morris-2016(BLCA)	WES	Based on clone numbers	Number of clones (Cutoff = 4)	I–IV	NA	359		1.05 (0.46,2.41)
20	Morris-2016(BRCA)	WES	Based on clone numbers	Number of clones (Cutoff = 2)	I–IV	NA	878		2.50 (1.12,5.20)
20	Morris-2016(HNSC)	WES	Based on clone numbers	Number of clones (Cutoff = 4)	I–IV	NA	280		3.75 (1.43,9.84)
20	Morris-2016(KIRC)	WES	Based on clone numbers	Number of clones (Cutoff = 5)	I–IV	NA	189		6.06 (1.85,19.85)
20	Morris-2016(LGG)	WES	Based on clone numbers	Number of clones (Cutoff = 4)	(–)	NA	484		8.30 (1.64,42.04)
20	Morris-2016(LUAD)	WES	Based on clone numbers	Number of clones (Cutoff = 4)	I–IV	NA	425		0.83 (0.40,1.74)
20	Morris-2016(LUSC)	WES	Based on clone numbers	Number of clones (Cutoff = 4)	I–IV	NA	178		1.59 (0.67,3.77)
20	Morris-2016(SKMC)	WES	Based on clone numbers	Number of clones (Cutoff = 4)	I–IV	NA	201		2.81 (0.96,8.25)
22	Mroz-2013(HNSC)	WES	Based on VAF directly	MATH (Cutoff: median)	I–IV	Single-region	39	39	2.46 (1.26,4.79)
21	Mroz-2015(HNSC)	WES	Based on VAF directly	MATH (Cutoff: MATH-value 32)	I–IV	NA	111	194	2.18 (1.44,3.30)
23	Obulkasim-2016(ESCA)-1	Array comparative genomic hybridization	Based on CNV	DNA copy number entropy (Cutoff: 33%)	I–III	Single-region	25	50	1.38 (1.01,1.88)
25	Pereira-2016(BRCA-ER-)	WES	Based on VAF directly	MATH (Cutoff: upper quartiles and lower quartiles)	NA	Multi-region	95	95	1.26 (0.81,1.95)
25	Pereira-2016(BRCA-ER+)	WES	Based on VAF directly	MATH (Cutoff: upper quartiles and lower quartiles)	NA	Multi-region	319	318	1.64 (1.23,2.20)
26	Schwarz-2015(OV)-1	WGS	Based on CNV	Clonal expansion (Cutoff: median)	I–IV	Multi-region	7	7	7.10 (>1.00)
27	Takaya-2020(OV)-1	WES	Based on clone numbers	Clonality Index (Cutoff: median)	I–IV	Multi-region	223	284	1.10 (0.85,1.41)
28	Turajlic-2018(KIRC)	WES	Based on VAF directly	ITH index (Cutoff: median ITH index value)	I–IV	NA	204	93	1.70 (1.00,2.70)
30	Yang-2019(COADREAD)-1	WES	Based on VAF directly	Subclonal mutations (Cutoff: receiver operating characteristic curves and the Youden index)	I–III	Single-region	23	5	35.44 (3.39,370.74)

VAF, variant allele frequency; CNV, copy number variation; ER, estrogen receptor; WGS, whole genome sequencing; WES, whole exome sequencing; MATH, mutant-allele tumor heterogeneity; HR, hazard ratio; CI, confidence interval; ITH, intratumor heterogeneity; NA, not available.

In general, these different ITH evaluation methods are essentially an integrated analysis of SNV, indels, and CNV, and the number of tumor subpopulations was simulated mathematically as the ITH value. Meanwhile, when different ITH assessment methods were used to analyze the same cancer type in TCGA (15, 20, 23, 24), the prognosis results showed consistency. Therefore, we pooled the results to generate an association between ITH and prognosis, even though some studies evaluated ITH differently.

### High ITH Was Associated With Worse Prognosis in General

**Figure 2:**
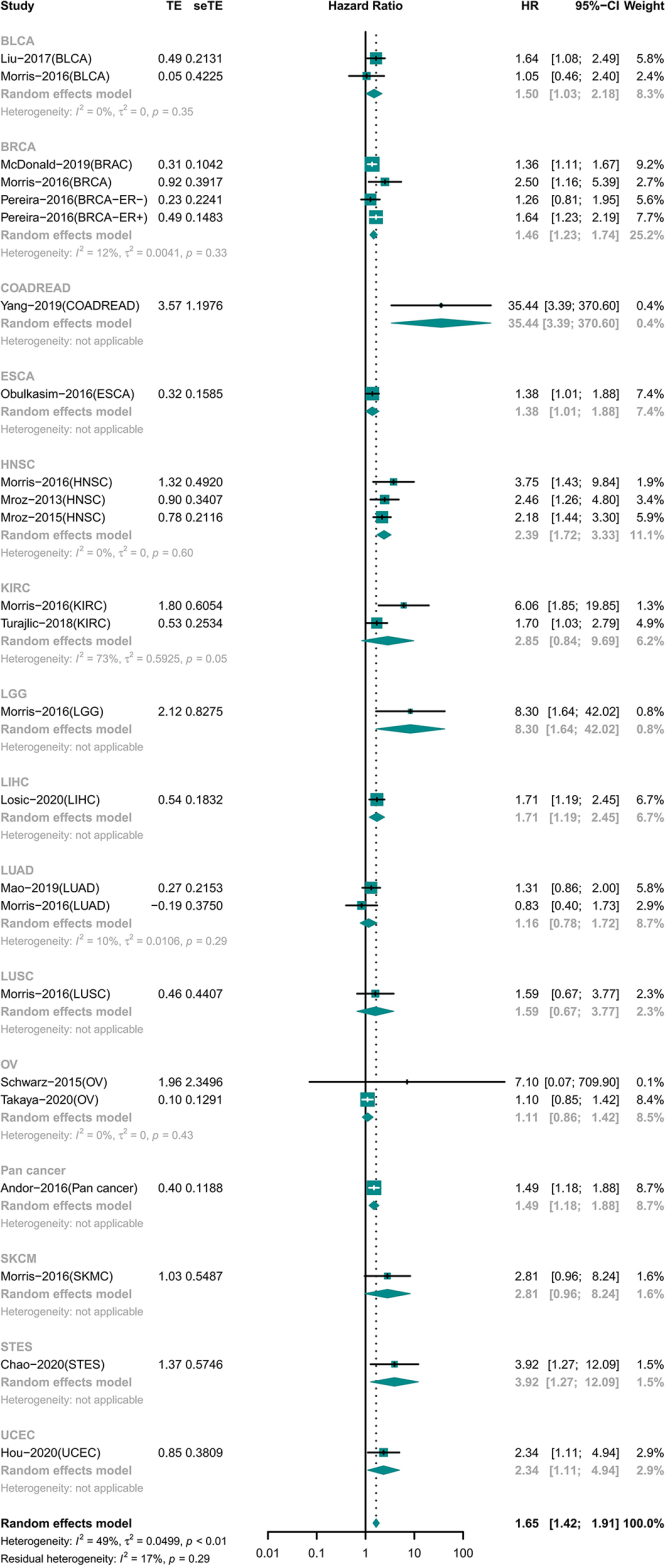
Forest plot of intratumor heterogeneity (ITH) with prognosis in OS.

A total of 7,971 participants involving 14 tumor types were examined for OS (15, 18–20, 22–26, 28–31, 33–35). The results showed that high ITH was associated with a high risk of death (HR 1.65 [95% CI, 1.42–1.91]) (**Figure 2**). However, this correlation was not homogeneous across all tumor types. Subgroup analysis showed that high ITH indicated a worse prognosis in patients with bladder urothelial carcinoma (BLCA), breast invasive carcinoma (BRCA), colon adenocarcinoma or rectum adenocarcinoma (COADREAD), esophageal carcinoma (ESCA), head and neck squamous cell carcinoma (HNSC), brain lower grade glioma (LGG), liver hepatocellular carcinoma (LIHC), gastroesophageal adenocarcinoma (STET), and uterine corpus endometrial carcinoma (UCEC). Conversely, ITH showed no correlation with prognosis in patients with kidney renal clear cell carcinoma (KIRC), lung adenocarcinoma (LUAD), lung squamous cell carcinoma (LUSC), ovarian serous cystadenocarcinoma (OV), and skin cutaneous melanoma (SKCM).

PFS was examined in 1,310 participants (17, 27, 29–31). Patients with high ITH were more likely to develop disease progression (HR 1.89 [95% CI, 1.41–2.54]) (**Supplementary Figure S1**). Subgroup analysis showed that high ITH was associated with poorer PFS for patients with KIRC, COADREAD, and OV.

In examining DFS, a total of 1,413 participants with five tumor types met the inclusion criteria (16, 17, 23, 31, 32). High ITH increased the risk of distant metastasis and recurrence (HR 1.87 [95% CI, 1.15–3.04]) (**Supplementary Figure S2**). We observed a correlation in patients with prostate adenocarcinoma (PRAD), COADREAD, LGG and glioblastoma multiforme (GBM), and thyroid carcinoma (THCA). Conversely, ITH showed no correlation with prognosis in patients with LUSC and LUAD.

### High ITH Indicated Short OS Independent of Distant Metastasis

**Figure 3:**
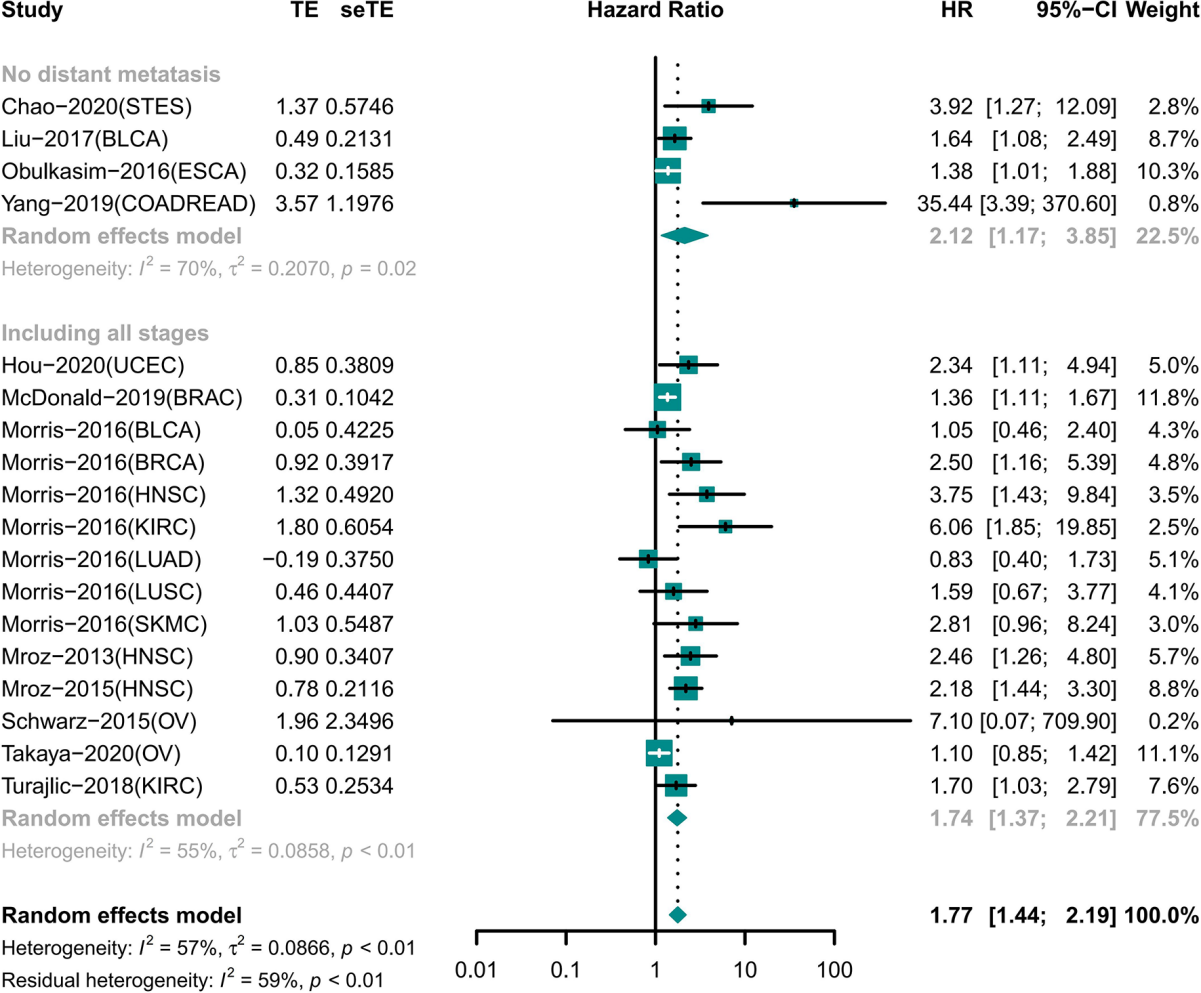
Forest plot of intratumor heterogeneity (ITH) with prognosis in different tumor stage.

A total of 18 cohorts involving 12 different tumor types recorded information for different tumor stages (**Figure 3**). Among the cohorts, 14 cohorts included patients with tumors of any stage involving nine tumor types; the results showed that the OS was shorter in the high ITH group (HR 1.74 [95% CI, 1.37–2.21]) (22–25, 29–31, 35). Four cohorts included information of four tumors with no distant metastasis, and high ITH was still associated with worse OS (HR 2.12 [95% CI, 1.17–3.85]) (18, 26, 33, 34).

### The Results of Single-Region and Multi-Region Sampling Were Similar for the Association of ITH and Prognosis

**Figure 4:**
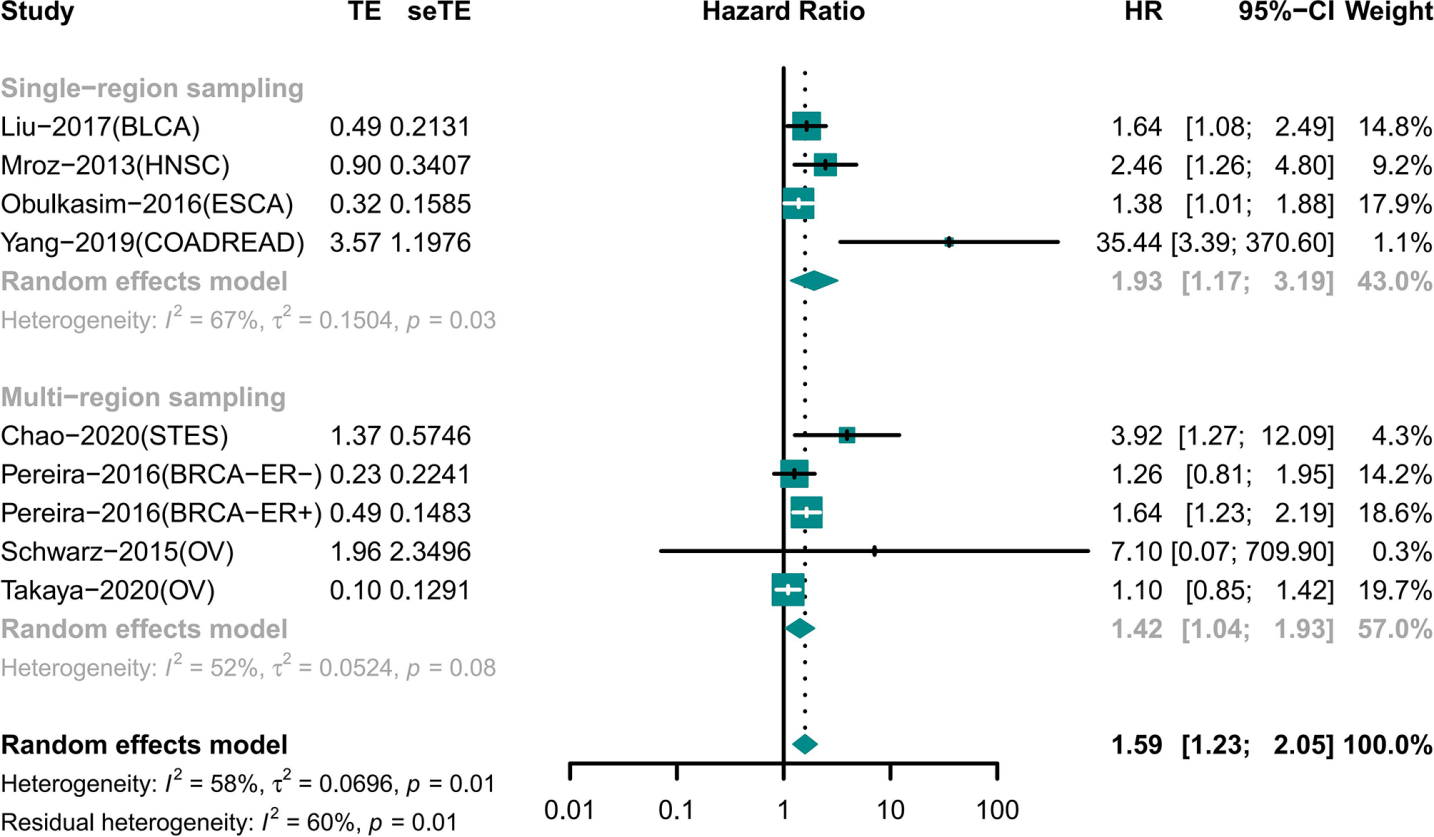
Forest plot of intratumor heterogeneity (ITH) with prognosis in diverse sampling model.

Night cohorts with precise descriptions of the sampling model were used to determine the impact of the sampling model on the results (**Figure 4**). These cohorts included patients with BLCA, BRCA, COADREAD, ESCA, HNSC, OV, and STES. Among the cohorts, four cohorts used single-region sampling and the analysis showed that high ITH patients had a worse prognosis (HR 1.93 [95% CI, 1.17–3.19]) (18, 25, 26, 33). Analysis of the other four cohorts using multi-region sampling indicated that patients with a high ITH had a higher risk of death (HR 1.42 [95% CI, 1.04–1.93]) (28–30, 34). The results of the two subgroups were similar.

### Publication Bias Analysis

Funnel plots for all forest maps are shown in **Supplementary Figure S3**.

## Discussion

This meta-analysis was an exploratory study probing the relationship between ITH and prognosis. Previous findings that examined the relation of ITH on prognosis have been controversial. The same is true for the prognostic implications of ITH for different tumor types. We demonstrated through a meta-analysis that a high ITH was associated with a poorer prognosis in patients with solid tumors. We stratified results by different solid tumors and found that the relation of ITH with prognosis was different for different tumor types. We also stratified results independently according to various confounding factors and found that various confounding factors did not affect the prognostic relation of ITH. Our findings have deepened the understanding of the field and suggest that ITH is a very promising indicator for clinical applications.

Overall, our analysis shows that OS, PFS, and DFS results indicate that the relationship between high ITH and worse prognosis is forceful. Seventeen solid tumors were included in these analyses. ITH was correlated with prognosis in 12 solid tumors. ITH may be the effect of time accumulation (36). Taking COADREAD as an example, the gradual progression from adenoma to adenocarcinoma contributes to the accumulation of ITH (37, 38), and the relation of ITH on prognosis may be more easily reflected in this cancer type. However, ITH was not associated with prognosis in patients with SKCM, KIRC, LUAD, LUSC, and OV in OS. Some tumors metastasize in the early stages (39, 40), and highly malignant tumors may have a shorter evolution time (41). ITH may not have enough time to develop to detect when tumor is diagnosed (41). Besides, because of the limitations of sequencing depth and purity of tumors, a large number of subclones may not be detected (42). In addition, various solid tumors can be further classified by pathological types. Although more research is needed to clarify the heterogeneity between tumor types, the available results are encouraging.

The challenge in ITH research is that there is no unified standard to calculate ITH, which greatly limits the clinical application. However, the vast majority of studies showed that there was no significant difference in the relationship between different ITH assessment methods and prognosis, regardless of single-region sampling or multi-region sampling. For example, in LUAD, different ITH evaluation methods [clone numbers (15), MATH (20), and subclonal populations (23)] show that ITH is not associated with prognosis. This may be because the principle of calculating the ITH is similar, which is based on SNV and CNV, and reflects the numbers of subpopulation in tumors. Of course, efforts have been made to develop more reasonable ITH algorithms, and a promising approach to evaluating ITH has been recently reported (43), Stefan et al. made a contribution to characterize ITH across cancer types based on the PCAWG dataset, including SNVs, indels, SVs, and CNVs, as well as subclonal drivers, subclonal selection, and mutation signatures. The assessment of prognosis based on the new method should be expected.

In the included studies, regardless of whether participants had distant metastasis, most of the samples used for sequencing were taken from the primary lesion. The correlation between ITH and prognosis was confirmed in tumors without distant metastasis. The increased number of subclones is associated with a greater possibility of distant metastasis, recurrence, and drug resistance, thus affecting the prognosis (44). However, for tumors in which distant metastases progress and more mutations appear, the results from primary tumor may not be sufficient. For patients with metastasis at the time of diagnosis, there may be differences in subclone composition between the metastatic lesion and primary lesion (45). Kim et al. proposed that the genetic distance between the metastatic and primary lesion may affect the prognosis (46). However, Reiter et al. (47) analyzed the ITH of distant metastases and found that CRC distant metastases are derived from subclones of the primary tumor. Anyway, so far, the results show that that detection in the primary lesion is sufficient to evaluate the role of ITH in prognosis.

Our results showed that single-site sampling and multi-region sampling were similar in terms of prognosis assessment. There was no evidence to suggest which sampling type was better because the subgroups did not come from the same cohorts. Huang et al. found that deep targeted sequencing of a single tumor specimen seems to be sufficient to evaluate ITH (48). However, tumor subclones have different spatial distribution (49). Zhang et al. reported that single-region sampling only reflects about 76% of the total mutation in multi-region sampling (50). Therefore, at present, when evaluating the ITH, sampling a single region may be sufficient in terms of validity or cost. Of course, if conditions permit, evaluating a diversified ITH provides more complete information.

It should be noted that these condition factors may not be perfectly matched. We analyzed the influence of different staging and sampling methods on the results based on all the information available in the original study, although this may somewhat damage the confidence level. The results presented by the available data are already suggestive; of course, more evidence is needed to verify our conclusions on staging and sampling methods in the future.

## Limitations

(1) Although the number of included cohorts and participants was large, the results did not cover all solid tumor types (2). Multiple cancer types and participant differences in the number of participants between TCGA cohort and small cohort studies lead to heterogeneity and publication bias risk in this study (3). For some tumor types, few cohorts were included, which was not enough to draw a positive or negative conclusion (4). The duration of follow-up varied from study to study; however, a minimum follow-up of 4 years is acceptable (5). Evaluation of ITH still requires a more uniform approach that could be better used in the clinic (6). Multiple confounders were analyzed in our meta-analysis. However, more strictly matching data are needed in the future to verify our conclusions (7). Some cancers (e.g., lung cancer) have shown that ITH is not prognostic and should be given more attention (8). For the moment, widespread clinical application could be a challenge. However, as tumor-based sequencing technologies become more widely available and ITH-related modeling algorithms continue to be developed, this problem is expected to be solved in the future.

## Conclusions

High ITH is associated with worse prognosis in many solid tumors in general, although this association was absent for some cancers. ITH is expected to be a promising clinical prognostic factor for the improvement of assessment, treatment, and surveillance strategy.

## Data Availability Statement

The original contributions presented in the study are included in the article/**Supplementary Material**. Further inquiries can be directed to the corresponding author.

## Author Contributions

TY: conceptualization and writing—original draft. XG: conceptualization and visualization, and writing—review and editing. SZ, ZZ, XZ, and CL: data curation. GL: conceptualization, project administration, supervision, and writing—review and editing. All authors contributed to the article and approved the submitted version.

## Conflict of Interest

The authors declare that the research was conducted in the absence of any commercial or financial relationships that could be construed as a potential conflict of interest.

## Publisher’s Note

All claims expressed in this article are solely those of the authors and do not necessarily represent those of their affiliated organizations, or those of the publisher, the editors and the reviewers. Any product that may be evaluated in this article, or claim that may be made by its manufacturer, is not guaranteed or endorsed by the publisher.
