# Supplementary material for: Intratumor Heterogeneity as a Prognostic Factor in Solid Tumors: A Systematic Review and Meta-Analysis
Source: Front Oncol. 2021 Oct 15;11:744064. doi: 10.3389/fonc.2021.744064 (PMC8554141; doi:10.3389/fonc.2021.744064)

Supplemental Material

eMethods Search strategy

(“Tumor heterogeneity” **OR** “Tumour heterogeneity” **OR** “Tumoral heterogeneity” **OR** “Tumoural heterogeneity” **OR** “Intratumor heterogeneity” **OR** “Intratumour heterogeneity” **OR** “Intratumoral heterogeneity” **OR** “Intratumoural heterogeneity” **OR** “Intertumor heterogeneity” **OR** “Intertumour heterogeneity” **OR** “Intertumoral heterogeneity” **OR** “Intertumoural heterogeneity” **OR** “Genetic heterogeneity” **OR** “Tumor evolution” **OR** “Evolution of tumor”)

AND

(Outcome **OR** Prognostic **OR** Prognosis **OR** Survival)

eTable 1 Included studies quality assessment

Study (First author, year)	1.Study participation	2.Study attrition	3.Prognostic factor measurement	4.Outcome measurement	5.Confounding measurement and account	6.Analysis	Quality grade
**Andor N et al.2016**	Yes	Yes	Yes	Yes	Yes	Yes	6
**Chao J al.2020**	Yes	Yes	Yes	Yes	Yes	Yes	6
**Hou Y al.2020**	Yes	Yes	Yes	Yes	Yes	Yes	6
**Jamal-Hanjani M et al.2017**	Yes	Yes	Yes	Yes	Yes	Yes	6
**Joung JG et al.2017**	Yes	Yes	Yes	Yes	Yes	Yes	6
**Liu D et al.2017**	Yes	Yes	Yes	Yes	Yes	Yes	6
**Losic B et al.2020**	Yes	Yes	Yes	Yes	No	Yes	6
**Mao H et al.2019**	Yes	Yes	Yes	Yes	No	Yes	6
**Masoodi T et al.2019**	Yes	Yes	Yes	Yes	Yes	Yes	6
**McDonald KA et al.2019**	Yes	Yes	Yes	Yes	Yes	Yes	6
**Morris LG et al.2016**	Yes	Yes	Yes	Yes	Yes	Yes	6
**Mroz EA et al.2013**	Yes	Yes	Yes	Yes	Yes	Yes	6
**Mroz EA et al.2015**	Yes	Yes	Yes	Yes	Yes	Yes	6
**Obulkasim A et al.2016**	Partly	Yes	Yes	Yes	Yes	Yes	5
**Oh BY et al.2019**	Yes	Yes	Yes	Yes	Yes	Yes	6
**Pereira B et al.2016**	Partly	Yes	Yes	Yes	No	Yes	4
**Schwarz RF et al.2015**	Yes	Yes	Yes	Yes	No	Yes	5
**Takaya H et al.2020**	Yes	Yes	Yes	Yes	Yes	Yes	6
**Turajlic S et al,2018**	Yes	Yes	Yes	Yes	No	Yes	5
**Wu P et al.2019**	Yes	Yes	Yes	Yes	Yes	Yes	6
**Yang J et al.2019**	Yes	Yes	Yes	Yes	No	Yes	5

eTable 2 Characteristics of all cohorts

Study(First author, year)	Country	Journal	Cohort name	Cancer	Detection
**Andor N et al.2016**	U.S.A.	Nat Med	**Andor-2016(Pan cancer)**	Pan cancer	WES
**Chao J al.2020**	U.S.A.	JAMA Netw Open	**Chao-2020(STES)**	Gastroesophageal adenocarcinoma	SNV Array Panel
**Hou Y al.2020**	China	Ann Transl Med	**Hou-2020(UCEC)**	Uterine corpus endometrial carcinoma	WES
**Jamal-Hanjani M et al.2017**	U.K.	N Engl J Med	**Jama-2017(LUAD&LUSC)**	Non Small Cell Lung Cancer	WES
**Joung JG et al.2017**	Korea	Clin Cancer Res	**Joung-2017(COADREAD)-1**	Colorectal cancer	WES
**Joung JG et al.2017**	Korea	Clin Cancer Res	**Joung-2017(COADREAD)-2**	Colorectal cancer	WES
**Liu D et al.2017**	U.S.A.	Nat Commun	**Liu-2017(BLCA)-1**	Chemotherapy resistant muscle-invasive bladder cancer	WES
**Liu D et al.2017**	U.S.A.	Nat Commun	**Liu-2017(BLCA)-2**	Chemotherapy resistant muscle-invasive bladder cancer	WES
**Liu D et al.2017**	U.S.A.	Nat Commun	**Liu-2017(BLCA)-3**	Chemotherapy resistant muscle-invasive bladder cancer	WES
**Losic B et al.2020**	U.S.A.	Nat Commun	**Losic-2020(LIHC)**	Hepatocellular carcinoma	WES
**Mao H et al.2019**	China	Ann Transl Med	**Mao-2019(LUAD)**	Lung adenocarcinoma	WES
**Masoodi T et al.2019**	Saudi Arabia	Am J Hum Genet	**Masoodi-2019(THCA)**	Papillary Thyroid Cancer	WES
**McDonald KA et al.2019**	U.S.A.	Ann Surg Oncol	**McDonald-2019(BRAC)**	Breast cancer	WES
**Morris LG et al.2016**	U.S.A.	Oncotarget	**Morris-2016(BLCA)**	Bladder urothelial carcinoma	WES
**Morris LG et al.2016**	U.S.A.	Oncotarget	**Morris-2016(BRCA)**	Breast invasive carcinoma	WES
**Morris LG et al.2016**	U.S.A.	Oncotarget	**Morris-2016(HNSC)**	Head and neck squamous cell carcinoma	WES
**Morris LG et al.2016**	U.S.A.	Oncotarget	**Morris-2016(KIRC)**	Clear cell carcinoma of the kidney	WES
**Morris LG et al.2016**	U.S.A.	Oncotarget	**Morris-2016(LGG)**	Lower grade glioma	WES
**Morris LG et al.2016**	U.S.A.	Oncotarget	**Morris-2016(LUAD)**	Lung adenocarcinoma	WES
**Morris LG et al.2016**	U.S.A.	Oncotarget	**Morris-2016(LUSC)**	Lung squamous cell carcinoma	WES
**Morris LG et al.2016**	U.S.A.	Oncotarget	**Morris-2016 (PRAD)**	Prostate adenocarcinoma	WES
**Morris LG et al.2016**	U.S.A.	Oncotarget	**Morris-2016(SKMC)**	Melanoma	WES
**Mroz EA et al.2013**	U.S.A.	Cancer-Am Cancer Soc	**Mroz-2013(HNSC)**	Head and neck squamous cell carcinoma	WES
**Mroz EA et al.2015**	U.S.A.	Plos Med	**Mroz-2015(HNSC)**	Head and neck squamous cell carcinoma	WES
**Obulkasim A et al.2016**	Netherlands	Oncotarget	**Obulkasim-2016(ESCA)-1**	Esophageal adenocarcinoma	Array comparative genomic hybridization

**Oh BY et al.2019**	Korea	Sci Rep-UK	**Oh-2019(COADREAD)**	Colorectal cancer	381 genes exons sequencing
**Pereira B et al.2016**	U.K.	Nat Commun	**Pereira-2016(BRCA-ER-)**	Breast cancer(ER-)	WES
**Pereira B et al.2016**	U.K.	Nat Commun	**Pereira-2016(BRCA-ER+)**	Breast cancer(ER+)	WES
**Schwarz RF et al.2015**	U.K.	PLoS Med	**Schwarz--2015(OV)-1**	High Grade Serous Ovarian Cancer	WGS
**Schwarz RF et al.2015**	U.K.	PLoS Med	**Schwarz--2015(OV)-2**	High Grade Serous Ovarian Cancer	WGS
**Takaya H et al.2020**	Japan	Gynecol Oncol	**Takaya-2020(OV)-1**	High-grade serous ovarian cancer	WES
**Takaya H et al.2020**	Japan	Gynecol Oncol	**Takaya-2020(OV)-2**	High-grade serous ovarian cancer	WES
**Turajlic S et al,2018**	U.K.	Cell	**Turajlic-2018(KIRC)**	Clear-cell renal cell carcinoma	WES
**Turajlic S et al,2018**	U.K.	Cell	**Turajlic-2018(KIRC)-TCGA**	Clear-cell renal cell carcinoma	WES
**Turajlic S et al,2018**	U.K.	Cell	**Turajlic-2018(KIRC)-TRAC**	Clear-cell renal cell carcinoma	WES
**Wu P et al.2019**	China	Oncol Lett	**Wu-2019(LGG+GBM)**	Malignant glioma	WES
**Yang J et al.2019**	China	Theranostics	**Yang-2019(COADREAD)-1**	Resistance to preoperative chemoradiotherapy rectal cancer	WES
**Yang J et al.2019**	China	Theranostics	**Yang-2019(COADREAD)-2**	Resistance to preoperative chemoradiotherapy rectal cancer	WES

eTable 2 Characteristics of all cohorts (Continue)

Cohort name	ITH assessment classification	ITH assessment details (Cut off)	Stage	Sampling	Treatment
**Andor-2016(Pan cancer)**	Based on clone numbers	Number of clones (Cut off = 2)	I-IV	NA	(-)
**Chao-2020(STES)**	Based on clone numbers	Number of clones (Cut off = 2)	I-III	Multi-region	(-)
**Hou-2020(UCEC)**	Based on VAF directly	MATH (Cut off: median)	I-IV	NA	(-)
**Jama-2017(LUAD&LUSC)**	Based on VAF directly	Subclonal mutations (Cut off: median)	I-III	Multi-region	(-)
**Joung-2017(COADREAD)-1**	Based on VAF directly	MATH (Cut off: median)	I-IV	Single-region	(-)
**Joung-2017(COADREAD)-2**	Based on clone numbers	Number of clones (Cut off = 2)	I-IV	Single-region	(-)
**Liu-2017(BLCA)-1**	Based on clone numbers	Number of clones (Cut off = 6)	I-III	Single-region	(-)
**Liu-2017(BLCA)-2**	Based on VAF directly	Subclonal mutations proportion (Cut off = 0.2)	I-III	Single-region	Surgery
**Liu-2017(BLCA)-3**	Based on VAF directly	Subclonal mutations proportion (Cut off = 20%)	I-III	Single-region	Surgery+Neoadjuvant chemotherapy
**Losic-2020(LIHC)**	Based on clone numbers	Number of clones (Cut off = 4)	NA	NA	(-)
**Mao-2019(LUAD)**	Based on VAF directly	MATH (Cut off: median)	NA	NA	(-)
**Masoodi-2019(THCA)**	Based on VAF directly	Subclonal mutations (Cut off: median)	I-III	Multi-region	(-)
**McDonald-2019(BRAC)**	Based on VAF directly	MATH(Cut off: median)	I-IV	NA	(-)
**Morris-2016(BLCA)**	Based on clone numbers	Number of clones (Cut off = 4)	I-IV	NA	(-)

**Morris-2016(BRCA)**	Based on clone numbers	Number of clones (Cut off = 2)	I-IV	NA	(-)
**Morris-2016(HNSC)**	Based on clone numbers	Number of clones (Cut off = 4)	I-IV	NA	(-)
**Morris-2016(KIRC)**	Based on clone numbers	Number of clones (Cut off = 5)	I-IV	NA	(-)
**Morris-2016(LGG)**	Based on clone numbers	Number of clones (Cut off = 4)	(-)	NA	(-)
**Morris-2016(LUAD)**	Based on clone numbers	Number of clones (Cut off = 4)	I-IV	NA	(-)
**Morris-2016(LUSC)**	Based on clone numbers	Number of clones (Cut off = 4)	I-IV	NA	(-)
**Morris-2016 (PRAD)**	Based on clone numbers	Number of clones (Cut off = 4)	I-IV	NA	(-)
**Morris-2016(SKMC)**	Based on clone numbers	Number of clones (Cut off = 4)	I-IV	NA	(-)
**Mroz-2013(HNSC)**	Based on VAF directly	MATH (Cut off: median)	I-IV	Single-region	(-)
**Mroz-2015(HNSC)**	Based on VAF directly	MATH (Cut off: MATH-value 32)	I-IV	NA	(-)
**Obulkasim-2016(ESCA)-1**	Based on CNV	DNA copy number entropy (Cut off: 33%)	I-III	Single-region	(-)
**Oh-2019(COADREAD)**	Based on VAF directly	Shannon's index (Cut off: Median)	I-IV	NA	(-)
**Pereira-2016(BRCA-ER-)**	Based on VAF directly	MATH (Cut off: upper quartiles and lower quartiles)	NA	Multi-region	(-)
**Pereira-2016(BRCA-ER+)**	Based on VAF directly	MATH (Cut off: upper quartiles and lower quartiles)	NA	Multi-region	(-)
**Schwarz--2015(OV)-1**	Based on CNV	Clonal expansion (Cut off: median)	I-IV	Multi-region	(-)
**Schwarz--2015(OV)-2**	Based on CNV	Clonal expansion (Cut off: median)	I-IV	Multi-region	(-)
**Takaya-2020(OV)-1**	Based on clone numbers	Clonality Index (Cut off: median)	I-IV	Multi-region	(-)
**Takaya-2020(OV)-2**	Based on clone numbers	Clonality Index (Cut off: median)	I-IV	Multi-region	(-)
**Turajlic-2018(KIRC)**	Based on VAF directly	ITH index (Cut off: median ITH index value)	I-IV	NA	(-)
**Turajlic-2018(KIRC)-TCGA**	Based on VAF directly	ITH index (Cut off: median ITH index value)	I-IV	NA	(-)
**Turajlic-2018(KIRC)-TRAC**	Based on VAF directly	ITH index (Cut off: median ITH index value)	I-IV	NA	(-)
**Wu-2019(LGG+GBM)**	Based on VAF directly	MATH (Cut off: median)	(-)	NA	(-)
**Yang-2019(COADREAD)-1**	Based on VAF directly	Subclonal mutations (Cut off: receiver operating characteristic curves and the Youden index)	I-III	Single-region	Pre-preoperative chemoradiotherapy
**Yang-2019(COADREAD)-2**	Based on VAF directly	Subclonal mutations (Cut off: receiver operating characteristic curves and the Youden index)	I-III	Single-region	Post-preoperative chemoradiotherapy

eTable 2 Characteristics of all cohorts (Continue)

Cohort name	Prognosis	Follow-up time (Month)	Participants (Low ITH)	Participants (High ITH)	Statistical method	HR(95%CI)
**Andor-2016(Pan cancer)**	OS	60	372	785	Log-Rank Test (Extracted)	1.49(1.18,1.88)
**Chao-2020(STES)**	OS	200	22	19	Log-Rank Test	3.92(1.27,12.08)
**Hou-2020(UCEC)**	OS	120+	121	121	Cox proportional hazards analysis	2.34(1.11,4.94)
**Jama-2017(LUAD&LUSC)**	DFS	30	49	51	Log-Rank Test	0.86(0.40,1.85)
**Joung-2017(COADREAD)-1**	PFS	120	59	29	Log-Rank Test (Extracted)	2.18(1.21,3.90)
**Joung-2017(COADREAD)-2**	DFS	120	27	61	Log-Rank Test (Extracted)	2.32(1.16,4.61)
**Liu-2017(BLCA)-1**	OS	60	15	15	Cox proportional hazards analysis	1.64(1.08,2.49)
**Liu-2017(BLCA)-2**	OS	60	16	14	Cox proportional hazards analysis	1.50(1.01,2.23)
**Liu-2017(BLCA)-3**	OS	60	16	14	Cox proportional hazards analysis	1.89(1.10,3.10)
**Losic-2020(LIHC)**	OS	120	85	102	Log-Rank Test (Extracted)	1.71(1.19,2.44)
**Mao-2019(LUAD)**	OS	60	115	115	Log-Rank Test (Extracted)	1.31(0.86,2.00)
**Masoodi-2019(THCA)**	DFS	60	41	38	Log-Rank Test	3.50(1.40,9.20)
**McDonald-2019(BRAC)**	OS	180	411	548	Log-Rank Test (Extracted)	1.36(1.11,1.67)
**Morris-2016(BLCA)**	OS	60	359		Cox proportional hazards analysis	1.05(0.46,2.41)
**Morris-2016(BRCA)**	OS	60	878		Cox proportional hazards analysis	2.50(1.12,5.20)
**Morris-2016(HNSC)**	OS	60	280		Cox proportional hazards analysis	3.75(1.43,9.84)
**Morris-2016(KIRC)**	OS	60	189		Cox proportional hazards analysis	6.06(1.85,19.85)
**Morris-2016(LGG)**	OS	60	484		Cox proportional hazards analysis	8.30(1.64,42.04)
**Morris-2016(LUAD)**	OS	60	425		Cox proportional hazards analysis	0.83(0.40,1.74)
**Morris-2016(LUSC)**	OS	60	178		Cox proportional hazards analysis	1.59(0.67,3.77)
**Morris-2016 (PRAD)**	DFS	60	389		Cox proportional hazards analysis	5.76(1.38,24.06)
**Morris-2016(SKMC)**	OS	60	201		Cox proportional hazards analysis	2.81(0.96,8.25)
**Mroz-2013(HNSC)**	OS	70	39	39	Log-Rank Test	2.46(1.26,4.79)
**Mroz-2015(HNSC)**	OS	40+	111	194	Cox proportional hazards analysis	2.18(1.44,3.30)
**Obulkasim-2016(ESCA)-1**	OS	120	25	50	Log-Rank Test	1.38(1.01,1.88)
**Oh-2019(COADREAD)**	PFS	60	152	152	Log-Rank Test (Extracted)	1.44(1.10,1.88)
**Pereira-2016(BRCA-ER-)**	OS	360	95	95	Log-Rank Test (Extracted)	1.26(0.81,1.95)
**Pereira-2016(BRCA-ER+)**	OS	360	319	318	Log-Rank Test (Extracted)	1.64(1.23,2.20)
**Schwarz--2015(OV)-1**	OS	52+	7	7	Log-Rank Test	7.10(>1.00)
**Schwarz--2015(OV)-2**	PFS	53+	7	7	Log-Rank Test	11.40(>1.00)
**Takaya-2020(OV)-1**	OS	120	223	284	Cox proportional hazards analysis	1.10(0.85,1.41)
**Takaya-2020(OV)-2**	PFS	120	223	284	Cox proportional hazards analysis	1.47(1.14,1.90)

**Turajlic-2018(KIRC)**	OS	120	204	93	Log-Rank Test	1.70(1.00,2.70)
**Turajlic-2018(KIRC)-TCGA**	PFS	120	204	93	Log-Rank Test	3.00(1.20,2.80)
**Turajlic-2018(KIRC)-TRAC**	PFS	60	53	47	Log-Rank Test	2.40(1.10,5.20)
**Wu-2019(LGG+GBM)**	DFS	60	387	370	Log-Rank Test	1.45(1.22,1.72)
**Yang-2019(COADREAD)-1**	OS	160	23	5	Cox proportional hazards analysis	35.44(3.39,370.74)
**Yang-2019(COADREAD)-2**	OS	160	16	12	Cox proportional hazards analysis	6.90(1.28,37.26)

VAF: variant allele frequency; CNV: copy number variation; ER: estrogen receptor; WGS: whole genome sequencing; WES: whole exome sequencing; MATH： mutant-allele tumor heterogeneity; HR: hazard ratio; CI: confidence interval; ITH: intratumor heterogeneity; NA: not available.

eFigure 1 Forest plot of intratumor heterogeneity (ITH) with prognosis in PFS

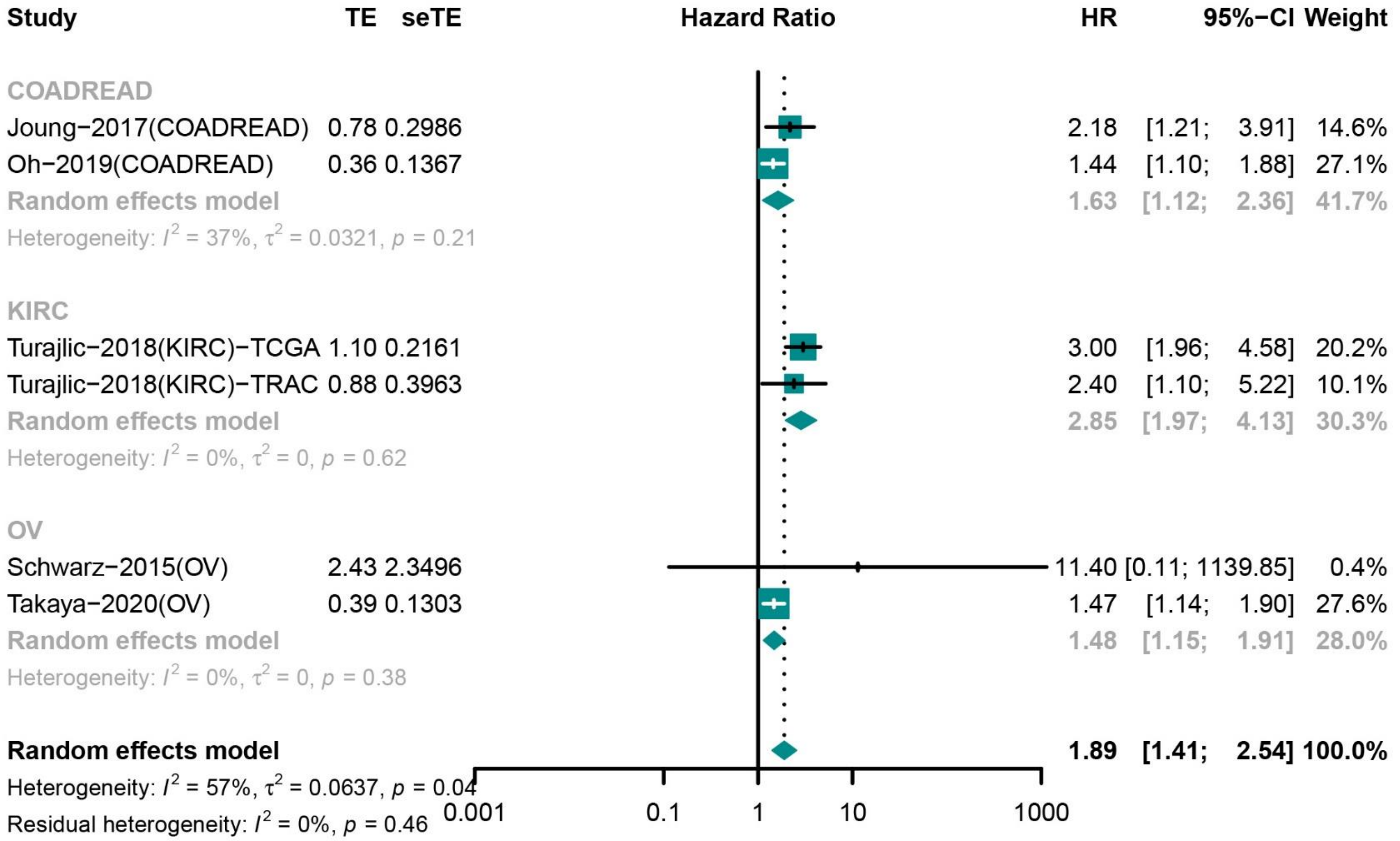

The point in the square reflects the hazard ratio, and the lines on each side reflect the 95% confidence interval. The square area reflects the weight of the cohorts. Diamonds indicate combination effects. The vertical dotted line reflects the combined HR. The vertical solid line reflects no association line. The left side of the no association line represents a low ITH, and the right side represents a high ITH.

eFigure 2 Forest plot of intratumor heterogeneity (ITH) with prognosis in DFS

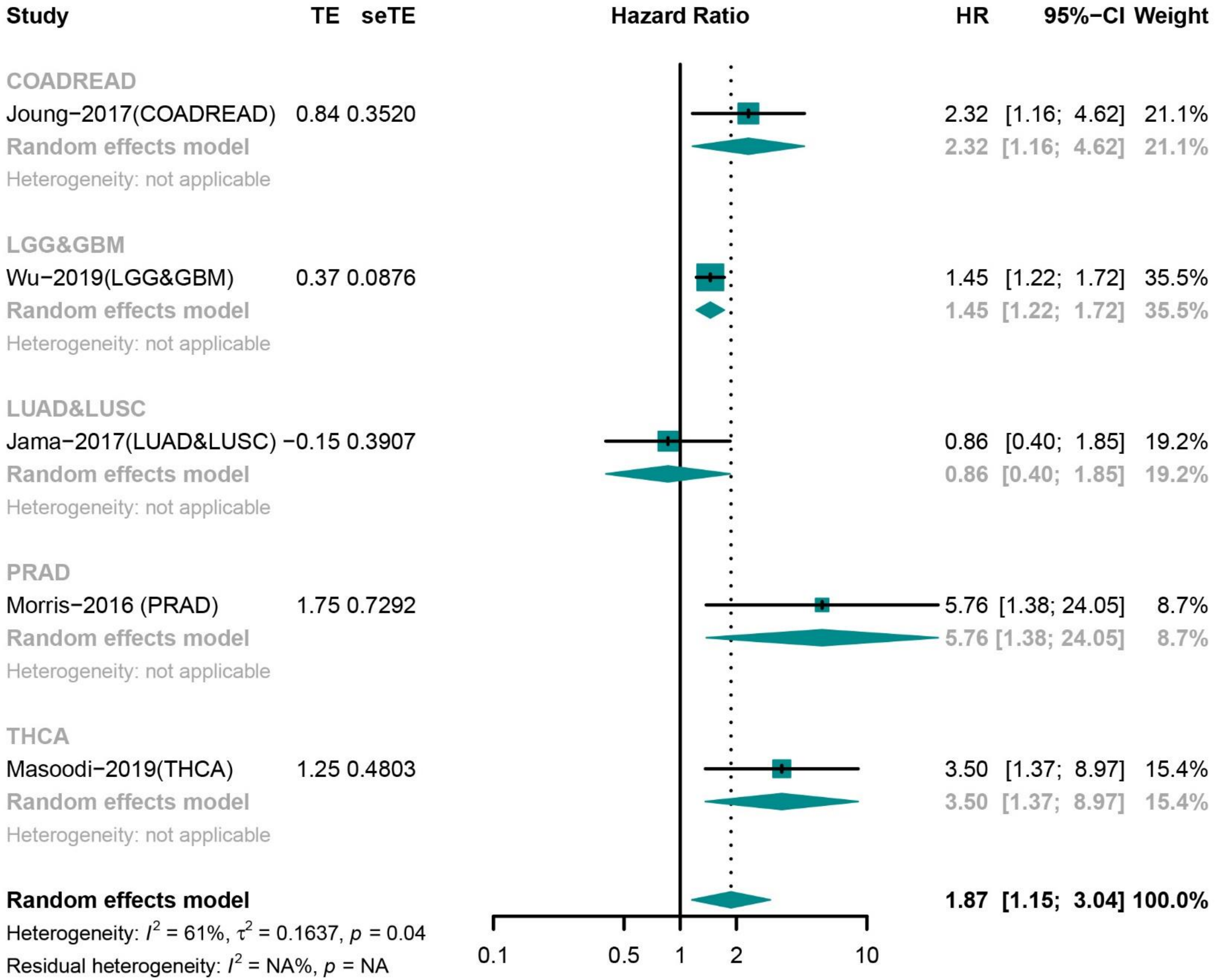

The point in the square reflects the hazard ratio, and the lines on each side reflect the 95% confidence interval. The square area reflects the weight of the cohorts. Diamonds indicate combination effects. The vertical dotted line reflects the combined HR. The vertical solid line reflects no association line. The left side of the no association line represents a low ITH, and the right side represents a high ITH.

eFigure 3 Funnel plot

eFigure 3-1 Funnel plot of Figure 2

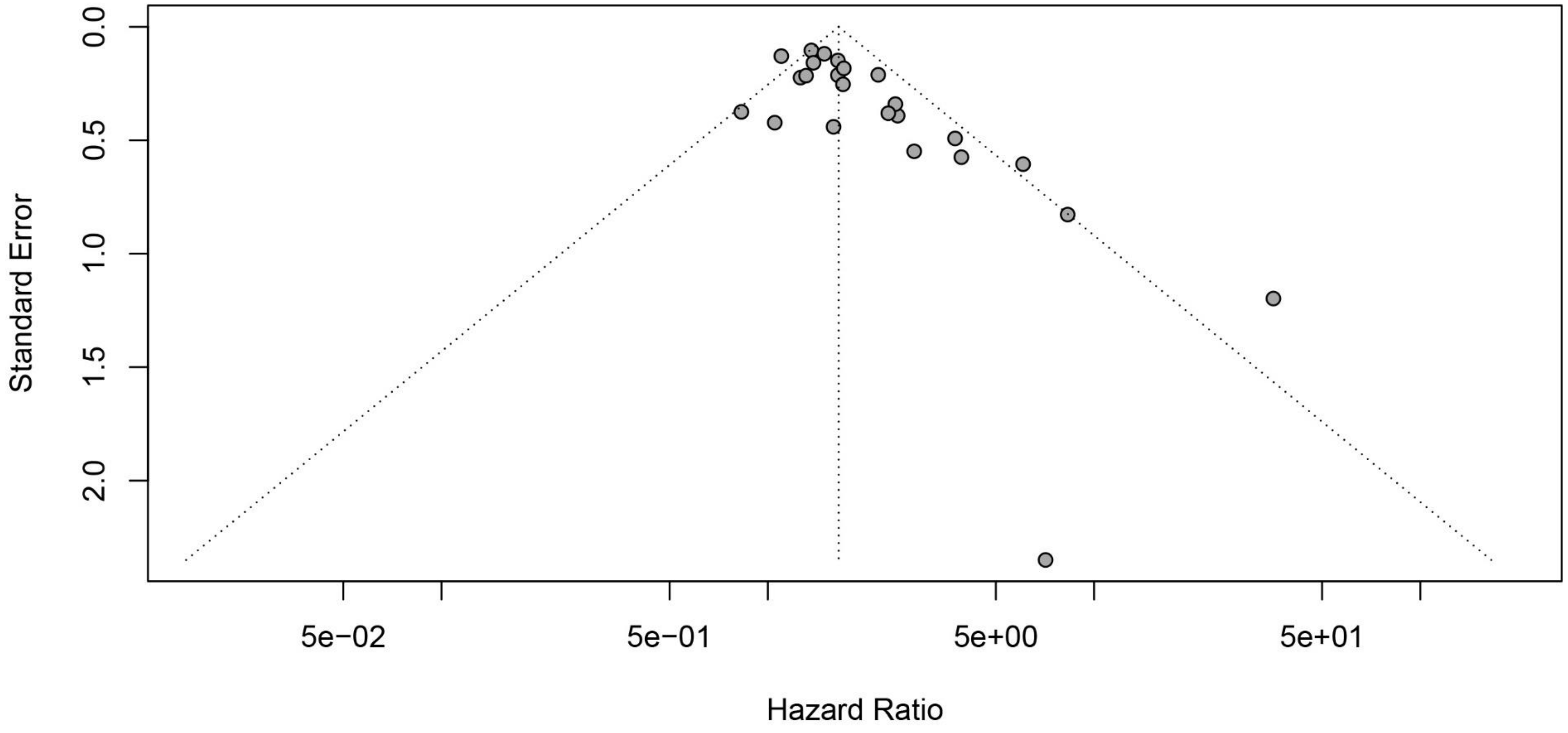

eFigure 3-2 Funnel plot of Figure 3

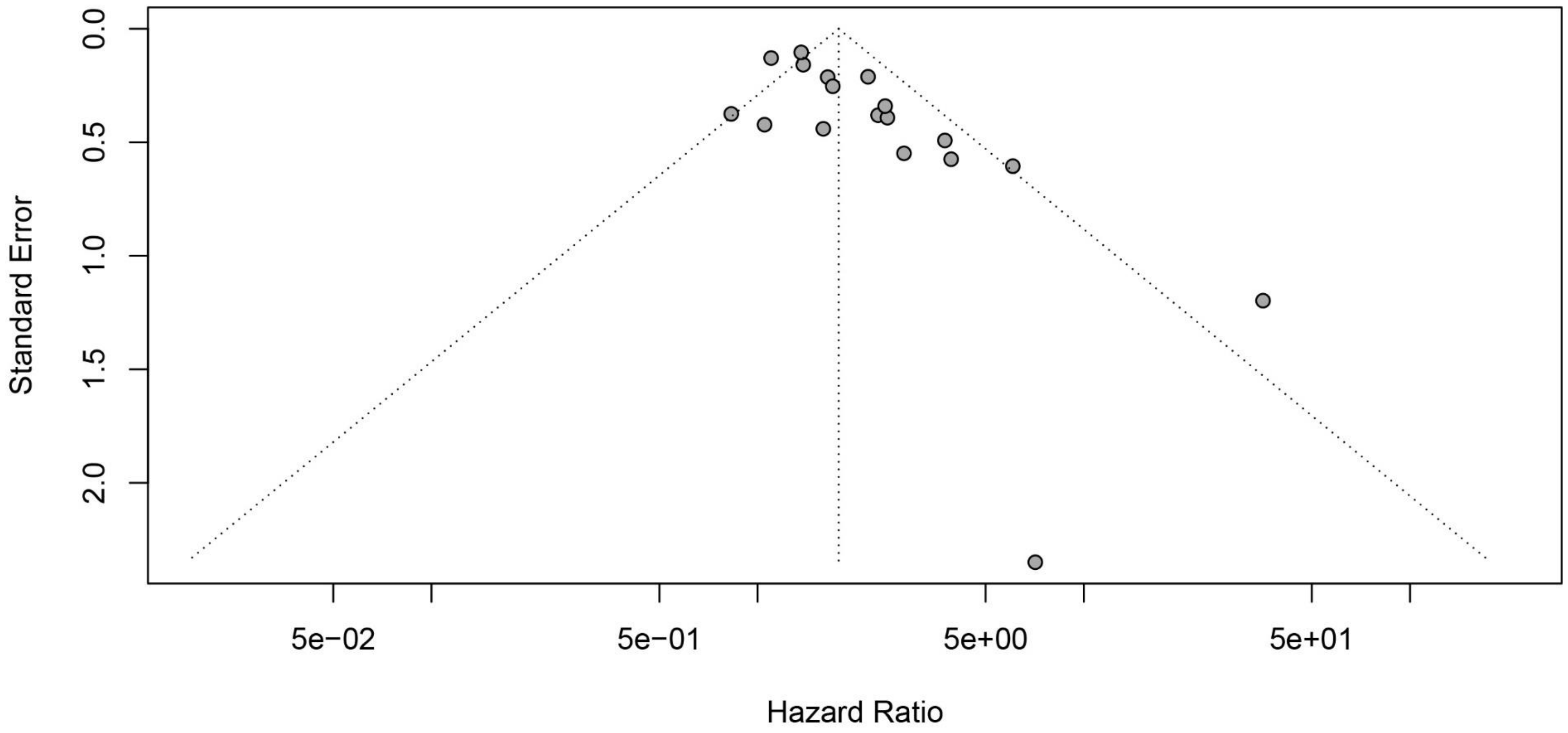

eFigure 3-3 Funnel plot of Figure 4

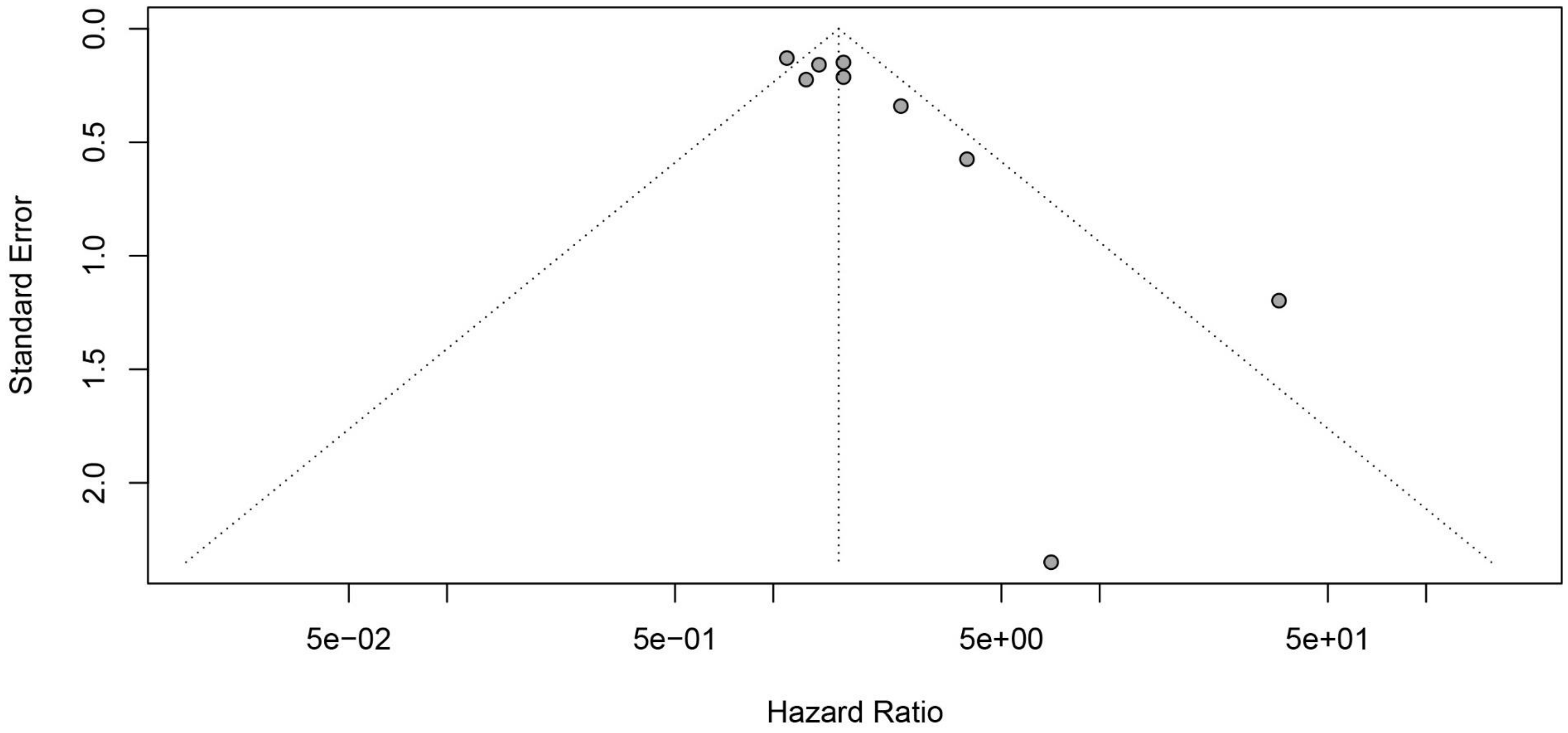

eFigure 3-4 Funnel plot of eFigure 1

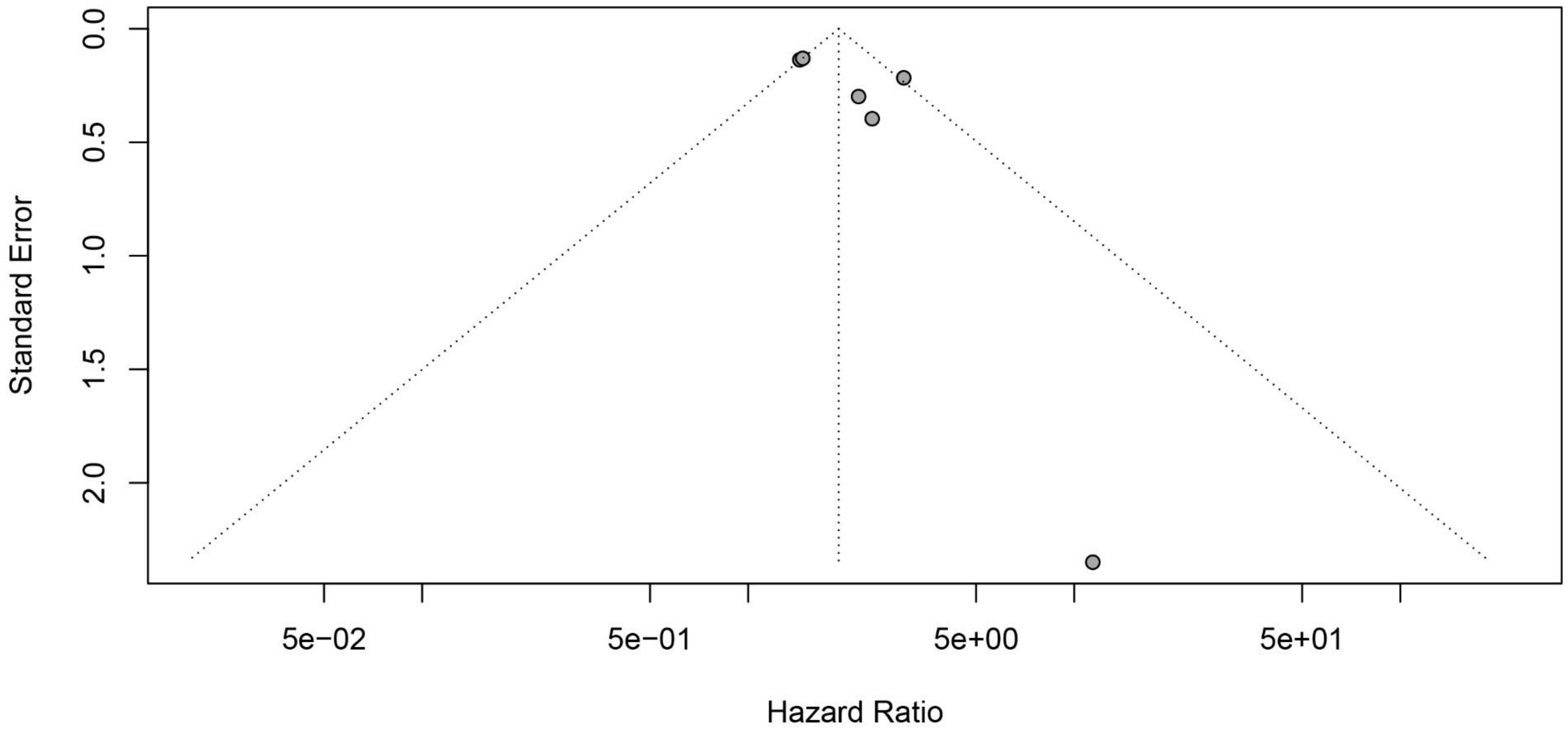

eFigure 3-5 Funnel plot of S eFigure 2

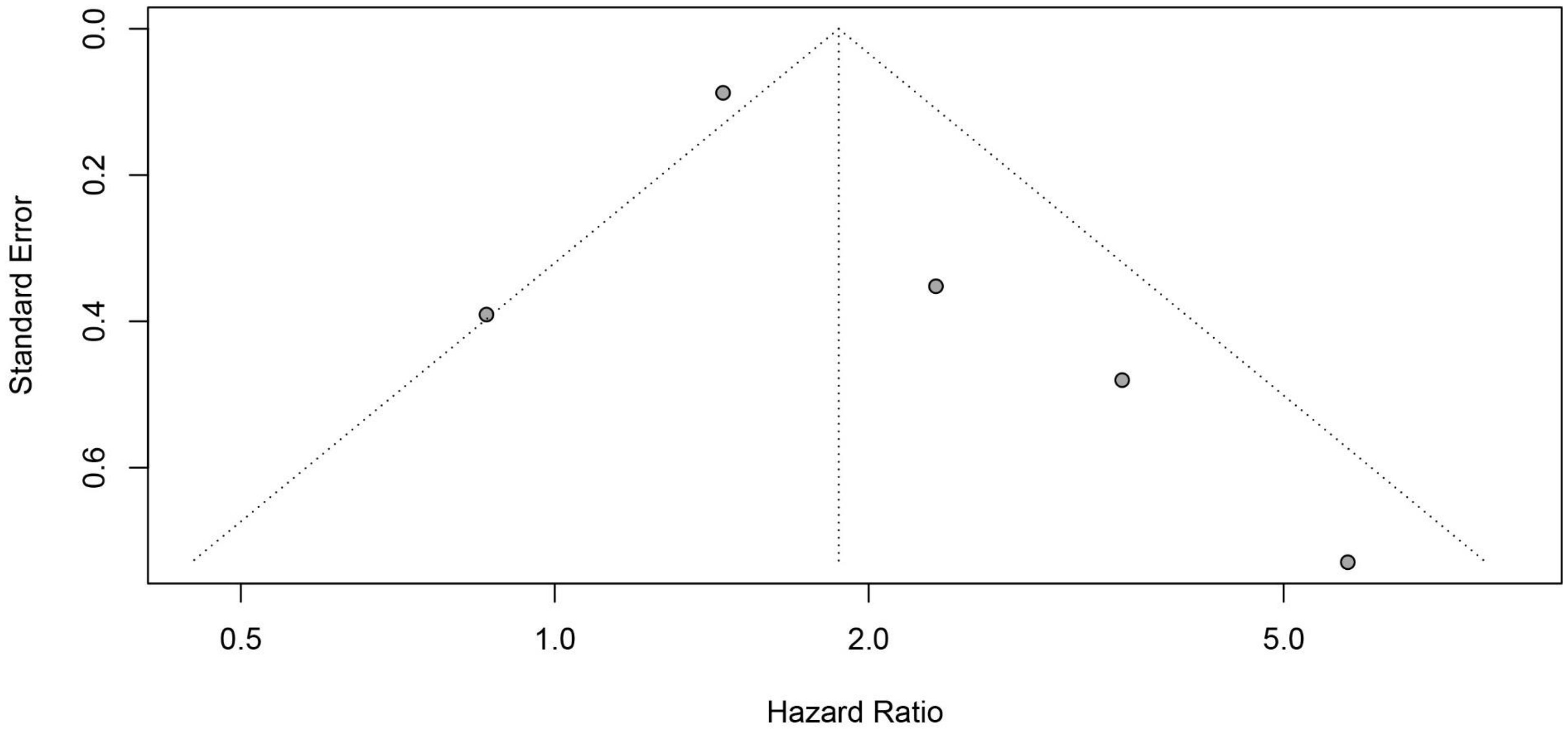

Supplement: Supplementary file 1 [file DataSheet_1.pdf]
